# Supplementary material for: Development and Consumer Perception of a Snack Machine Producing Customized Spoonable and Drinkable Products Enriched in Dietary Fiber and Protein
Source: Foods. 2020 Oct 13;9(10):1454. doi: 10.3390/foods9101454 (PMC7601953; doi:10.3390/foods9101454)
Supplement: Supplementary file 1 [file foods-09-01454-s001.pdf]

Development and consumer perception of a snack machine producing customized spoonable and drinkable products enriched in dietary fiber and protein

Supplementary materials:

Supplementary data, Table S1: List of sensory attributes and their reference products in the sensory profiling of drinkable and spoonable cocoa-flavored samples. a: D: drinkable, S: spoonable.

Supplementary data, Figure S1: Screenshots of the functional user interface of the HSM (PAGE 5-9). Scoothie wording was used in the user interface as a snack product name. (A)Screenshot of the functional user-interface of the HSM, starting page; (B) Screenshot of the functional user-interface of the HSM, suggestions included six pre-prepared options with fiber (F). (C) Screenshot of the functional user-interface of the HSM, 'create your own' route, selection of consistency, base and flavor. (D) Screenshot of the functional user-interface of the HSM, 'create your own' route, selection of protein, fiber and garnish. (E) Screenshot of the functional user-interface of the HSM, 'create your own' route, nutritional values and selection of the size

Supplementary data, Table S2: were related to HSM testing itself, and questions 15-17 were the background questions.

Supplementary data, Figure S2: Sensory profiling for drinkable and spoonable samples. (A) Spider plot of the sensory profile of drinkable cocoa-flavored samples. The plot is drawn using average values of 10 assessors and two replicate evaluations. (B) Spider plot

of the sensory profile of spoonable cocoa-flavored samples. The plot is drawn using average values of 10 assessors and two replicate evaluations. (PAGE 8)

Supplementary data, Figure S3: Preferred base and size of the products. (A) Consumers' willingness to buy the samples in the coming month from HSM as they were presented in the consumer test. 1 = very unlikely to buy, 7 = very likely to buy. There was no significant association between sample type and willingness to buy. (B) Consumers' preferred portion sizes for the cocoa-flavored samples. There was no significant association between sample type and preferred portion size. (PAGE 9)

Supplementary data Table S1.

Table S1. List of sensory attributes and their reference products in the sensory profiling of drinkable and spoonable cocoa-flavored samples. a: D: drinkable, S: spoonable.

Attribute	*Sample types (a)*	*Description*	*Reference product*	*Intensity*
Odor				
Cocoa/Chocolate	D, S	Odor reminiscent of cocoa powder, chocolate or a chocolate beverage	Cocoa drink made on skim milk (1.5% fat, Valio Oy, Helsinki, Finland) containing 2.5% Van Houten cocoa powder (Barry Callebaut Belgium NV, Lebberke-Wieze, Belgium) and 2.5% sucrose (Suomen Sokeri Oy, Kantvik, Finland). A 40 ml portion was served in a closed plastic cup.	6
Sweet	D	Sweet odor		7
Cereal-like/oat-like	D, S	Odor reminiscent of oat groats or a cereal field	10% solution of oat powder (Puhtikaura, Viipurilainen kotileipomo Oy, Vääksy, Suomi) in water.	6
Dried pea	D, S	Odor reminiscent of dried peas or beans	Halved yellow peas (Arolan tila, Pornainen, Finland) that were heated in a microwave oven. An 8 g portion was served in a closed plastic cup.	7
Appearance and texture				
Sedimentation tendency	D	Tendency of the sample to separate into two phases 20 seconds after mixing	None	
Ropiness (with a spoon)	S	Flowing tendency of the sample when pouring from the spoon; ranges between viscous clumps to long ropy threads	Rye porridge made by mixing coarse and fine rye flour in 1:4 ratio to a 15% suspension in water. The solution was boiled for 1 minute and cooled to	2

			ambient temperature. A 40 ml portion was served in a closed plastic cup.	
			Oat powder solution (as above)	7
Consistency (spoonable)	S	Ability of the sample to resist moving around with a spoon	Rye porridge (as above)	5
Graininess	S	The number of hard particles felt during chewing	Oat powder solution (as above)	1
			Rye porridge (as above)	6
Sliminess	S	Feeling of a slippery coating caused by the sample in the mucous membrane of the mouth	Oat powder solution (as above)	9
Taste and flavor				
Sweetness	D, S	Sweet taste	Cocoa drink (as above)	7
Cocoa/Chocolate	D, S	Flavor reminiscent of cocoa powder, chocolate or a chocolate beverage		7
Bitterness	D, S	Bitter taste	Oat powder solution (as above)	7
Cereal-like/oat-like	D, S	Flavor reminiscent of oat groats or a cereal field		7
Dried pea	D, S	Flavor reminiscent of dried peas or beans	Pea reference (as above); flavor intensity was compared to the odor intensity of the reference.	8
Possible other flavor intensity	D, S	Intensity of other flavors apart from the attribute list	None	
Mouthfeel				
Astringency	D	Mouth drying, puckering or shrinking sensation in the mouth	Oat powder solution (as above)	5
Richness/creaminess	D, S	Sensation of cream-like or velvety	None	

Supplementary data Figure S1.

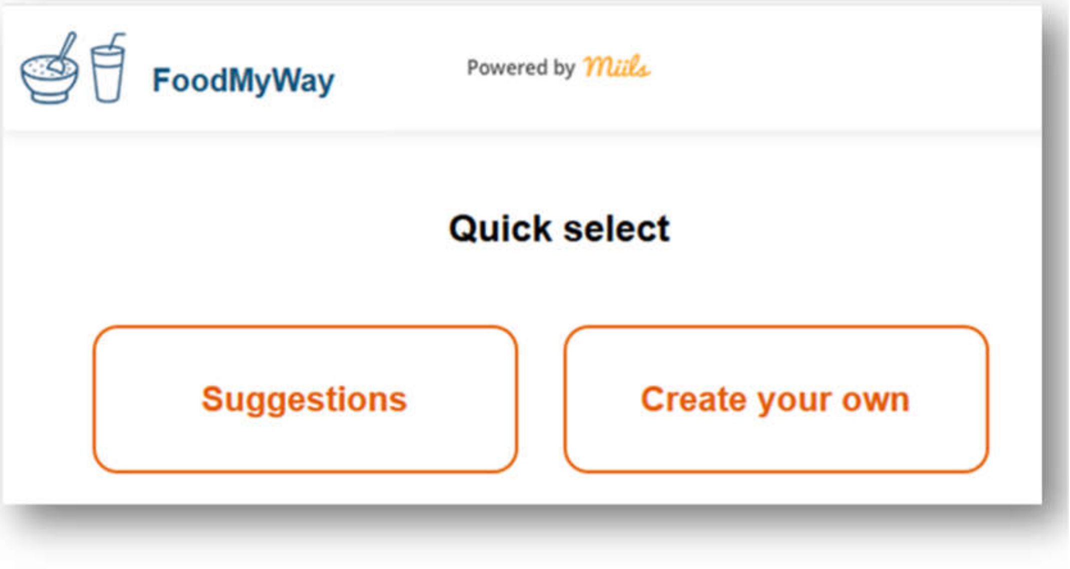

(A)

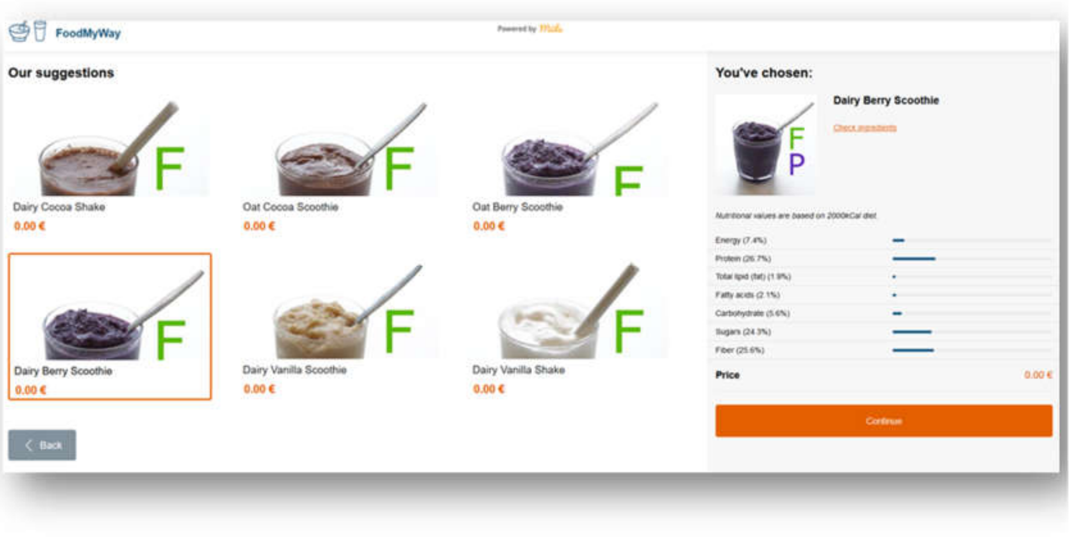

(B)

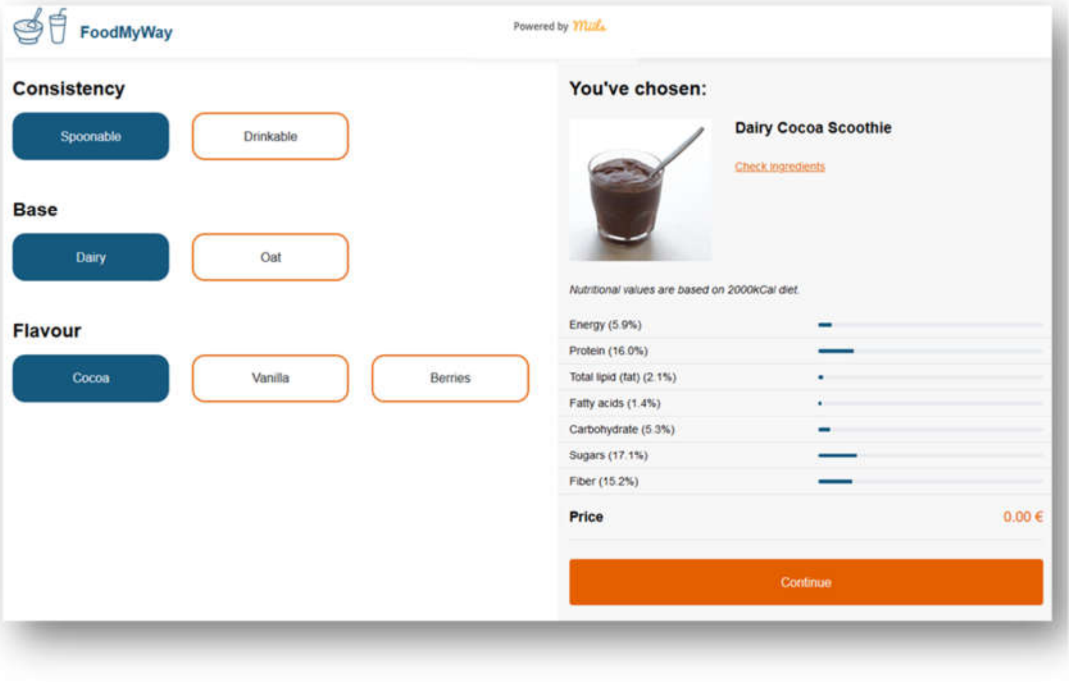
FoodMyWay
Powered by
Consistency
Spoonable
Drinkable
Base
Dairy
Oat
Flavour
Cocoa
Vanilla
Berries
You've chosen:
Dairy Cocoa Scoothie
Check ingredients
Nutritional values are based on 2000kCal diet.
Energy (5.9%)
Protein (16.0%)
Total lipid (fat) (2.1%)
Fatty acids (1.4%)
Carbohydrate (5.3%)
Sugars (17.1%)
Fiber (15.2%)
Price
0.00 €
Continue

(C)

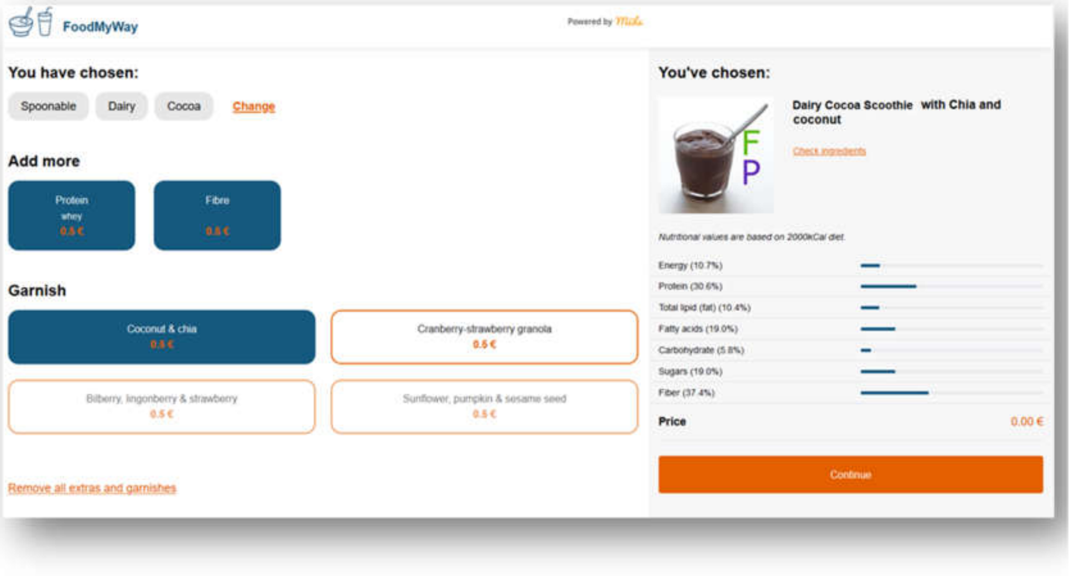
FoodMyWay
You have chosen:
Spoonable
Dairy
Cocoa
Change
Add more
Protein whey
0.5 €
Fibre
0.5 €
Garnish
Coconut & chia
0.5 €
Cranberry-strawberry granola
0.5 €
Bilberry, lingonberry & strawberry
0.5 €
Sunflower, pumpkin & sesame seed
0.5 €
Remove all extras and garnishes
You've chosen:
Dairy Cocoa Scoothie with Chia and coconut
Check ingredients
Nutritional values are based on 2000kCal diet.
Energy (10.7%)
Protein (30.6%)
Total lipid (fat) (10.4%)
Fatty acids (19.0%)
Carbohydrate (5.8%)
Sugars (19.0%)
Fiber (37.4%)
Price
0.00 €
Continue

(D)

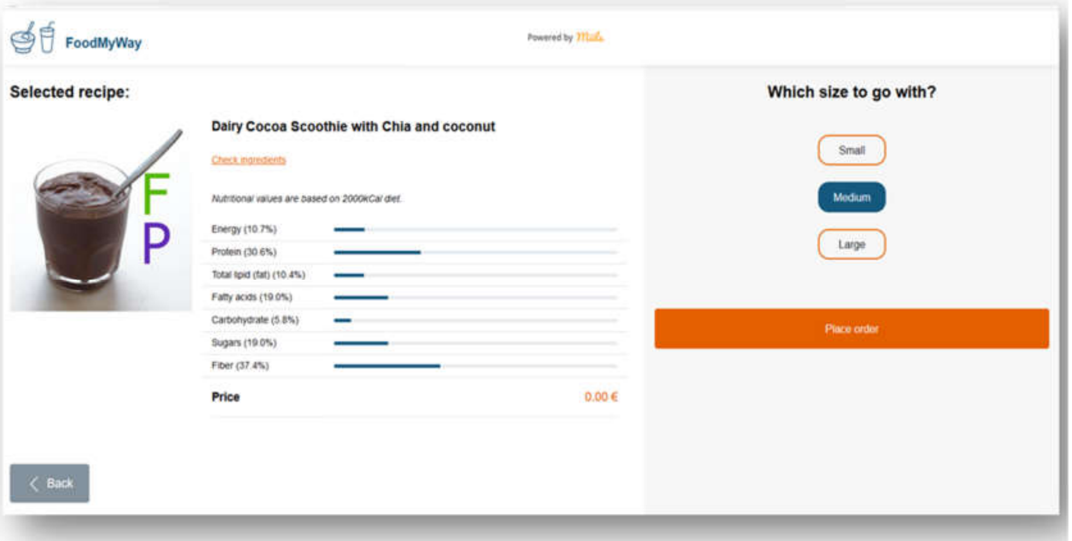

(E

Figure S1. Screenshots of the functional user interface of the HSM (PAGE 5-9). Scoothie wording was used in the user interface as a snack product name. (**A**)Screenshot of the functional user-interface of the HSM, starting page; (**B**) Screenshot of the functional user-interface of the HSM, suggestions included six pre-prepared options with fiber (F). (**C**) Screenshot of the functional user-interface of the HSM, 'create your own' route, selection of consistency, base and flavor. (**D**) Screenshot of the functional user-interface of the HSM, 'create your own' route, selection of protein, fiber and garnish. (**E**) Screenshot of the functional user-interface of the HSM, 'create your own' route, nutritional values and selection of the size.

Supplementary data Table S2.

Table S2. were related to HSM testing itself, and questions 15-17 were the background questions.

No	Question	Options
1	What kind of product did you choose?	Self-tailored product<br>Quick choice 1<br>Quick choice 2<br>Quick choice 3
2	The tailored product was…	Drinkable<br>Spoonable
3	The tailored product was…	Dairy-based<br>Oat-based<br>Can't remember
4	The tailored product was…	Cocoa-flavored<br>Vanilla-flavored<br>Berry-flavored
5	Which of the following extras did you choose?	Extra protein<br>Extra fiber<br>Coconut & chia<br>Almond and strawberry muesli<br>Can't remember
6	Which product size did you choose?	S (1 dl)<br>M (2 dl)<br>L (3 dl)
7	How else would you want to tailor the product or its extras?	(Open question)
8	Evaluate the user experience of the machine and the product you received based on the following statements<br>• The machine was easy to use<br>• The UI of the machine was clear<br>• I was able to order the product I wanted easily<br>• I was able to order the product I wanted quickly<br>• I received enough information about the nutritional content of the product I chose<br>• I was able to find an option that I found desirable<br>• The product I received looked appealing<br>• The product I received smelled appealing<br>• The product I received tasted good<br>• The product selection was large enough<br>• There were enough tailoring options available<br>• The product I ordered seemed healthy<br>• My product was prepared quickly enough<br>• The product I received met my expectations<br>• The product took away hunger<br>• The product took away thirst	1 - Strongly disagree<br>2 - Disagree<br>3 - Can't say<br>4 - Agree<br>5 - Strongly agree
9	How interested would you be in buying products from a machine like this in the future? Which factors have an effect on your interest?	(Open question)
10	In which situations would you imagine yourself using a machine like this?	(Open question)

11	What do you think would be a good location for the machine?	(Open question)
12	How much would you be willing to pay for a 2 dl portion? If you want, you can justify your answer.	(Open question)
13	What do you think would be a good portion size?	1 dl<br>2 dl<br>3 dl
14	How do you think the product concept you tested should be developed further?	(Open question)
15	Gender	Male<br>Female<br>Other<br>Don't want to say
16	Age	18 - 29<br>30 - 39<br>40 - 49<br>50 - 59<br>Over 60<br>Don't want to say
17	Choose the options below that best describe your life:<br>• I eat breakfast<br>• I eat lunch<br>• I eat a snack during the workday<br>• I bring a packed lunch / snack to the workplace from home<br>• I use the vending machine at the workplace<br>• I go to the nearby grocery store during the workday to buy something to eat<br>• I eat snack products (cookies, bars, smoothies…)	Once a year or less<br>A couple of times a year<br>1 - 3 times a month<br>Once a week<br>2 - 4 times a week<br>Once a day or more often

Supplementary data Figure S2.

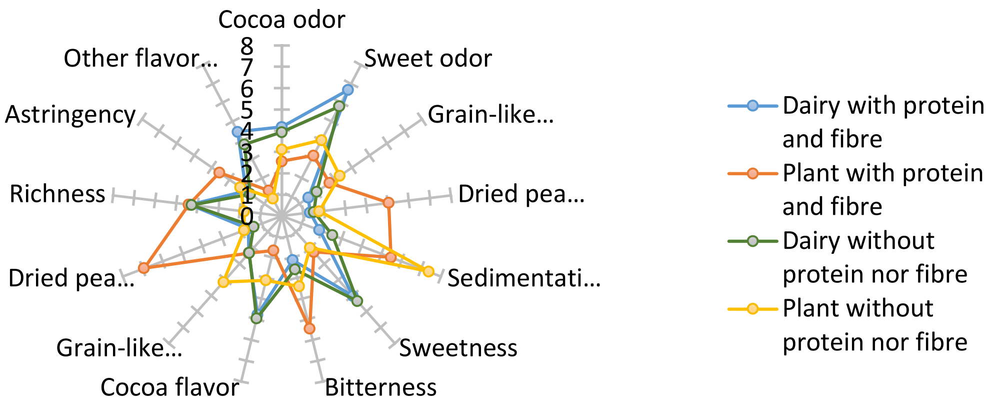

(A)

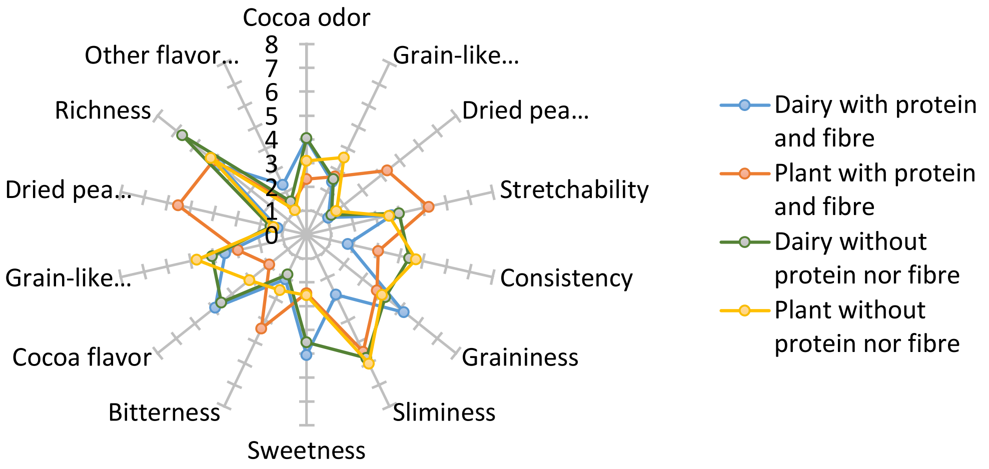

(B)

Figure S2. Sensory profiling for drinkable and spoonable samples. (**A**) Spider plot of the sensory profile of drinkable cocoa-flavored samples. The plot is drawn using average values of 10 assessors and two replicate evaluations. (**B**) Spider plot of the sensory profile of spoonable cocoa-flavored samples. The plot is drawn using average values of 10 assessors and two replicate evaluations.

Supplementary data Figure S3.

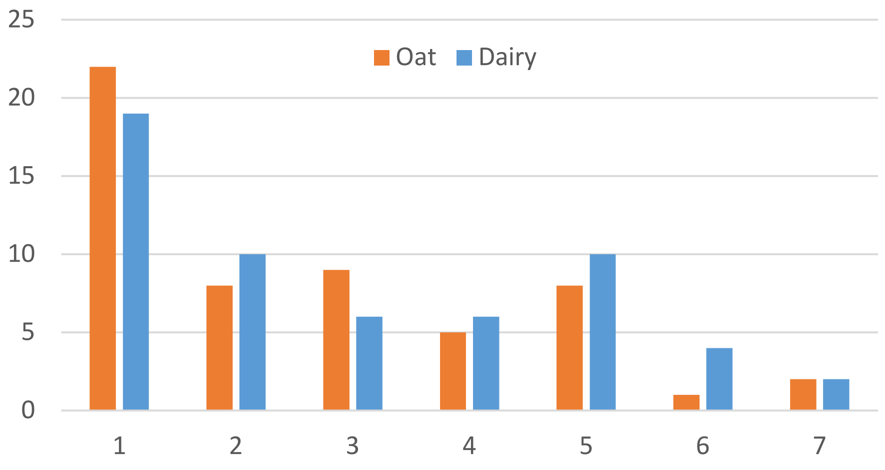

(A)

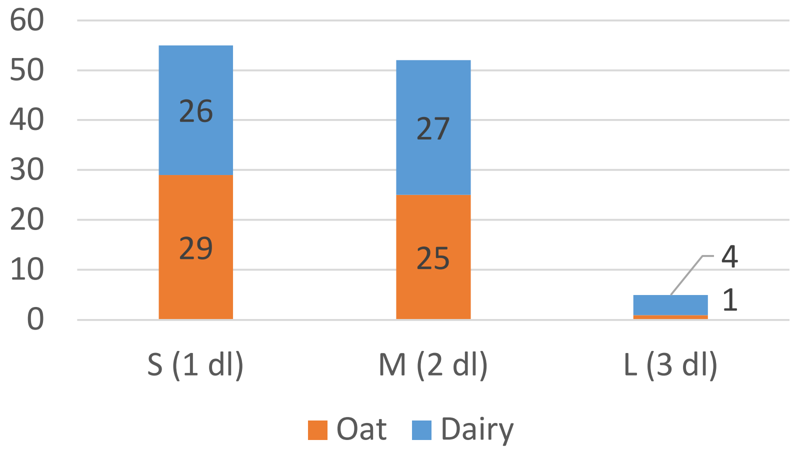

(B)

Figure S3. Preferred base and size of the products. (**A**) Consumers' willingness to buy the samples in the coming month from HSM as they were presented in the consumer test. 1 = very unlikely to buy, 7 = very likely to buy. There was no significant association between sample type and willingness to buy. (**B**) Consumers' preferred portion sizes for the cocoa-flavored samples. There was no significant association between sample type and preferred portion size.
